# Body Composition, Dietary Intake and the Risk of Low Energy Availability in Elite-Level Competitive Rhythmic Gymnasts

**DOI:** 10.3390/nu13062083

**Published:** 2021-06-18

**Authors:** María Villa, José G. Villa-Vicente, Jesus Seco-Calvo, Juan Mielgo-Ayuso, Pilar S. Collado

**Affiliations:** 1 Department of Health Sciences, Faculty of Health Sciences, University Pontificia of Salamanca, 37002 Salamanca, Spain; mvillabo.mag@upsa.es; 2 Institute of Biomedicine (IBIOMED), University of Leon, 24071 Leon, Spain; jg.villa@unileon.es (J.G.V.-V.); mpsanc@unileon.es (P.S.C.); 3 Department of Physiology, University of the Basque Country, 48940 Leioa, Spain; 4 Department of Health Sciences, Faculty of Health Sciences, University of Burgos, 09001 Burgos, Spain; jfmielgo@ubu.es

**Keywords:** elite rhythmic gymnasts, nutritional intake, body composition, energy availability

## Abstract

The aim of this study was to analyze dietary intake and body composition in a group of elite-level competitive rhythmic gymnasts from Spain. We undertook body composition and nutritional analysis of 30 elite gymnasts, divided into two groups by age: pre-teen (9–12 years) (*n* = 17) and teen (13–18 years) (*n* = 13). Measures of height, weight, and bioimpedance were used to calculate body mass index and percent body fat. Energy and nutrient intakes were assessed based on 7-day food records. The two groups had similar percentages of total body fat (pre-teen: 13.99 ± 3.83% vs. teen: 14.33 ± 5.57%; *p* > 0.05). The energy availability values for pre-teens were above the recommended values (>40 kcal/FFM/day) 69.38 ± 14.47 kcal/FFM/day, while those for the teens were much lower (34.7 ± 7.5 kcal/FFM/day). The distribution of the daily energy intake across the macronutrients indicates that both groups ingested less than the recommended level of carbohydrates and more than the recommended level of fat. Very low intakes of calcium and vitamin D among other micronutrients were also noted. The main finding is that teenage gymnasts do not consume as much energy as they need each day, which explains their weight and development. Moreover, they are at a high risk of developing low energy availability that could negatively impact their performance and future health.

## 1. Introduction

Training and competing at professional levels require optimal body function, which relies on adequate dietary intake providing sufficient energy as well as macro- and micronutrients [1]. It is widely recognized that elite gymnasts follow intense training routines from a young age, specifically, 7- to 18-year-old girls may be subjected to training loads of as high as 21–37 h a week for 11–12 months a year [2,3]. Growing numbers of authors are expressing concerns that intense training from an early age is responsible for delays in the growth, maturation, and body composition of young female athletes in general and elite gymnasts in particular, as they tend to have started gymnastics very young (5 to 6 years old) [4,5] and continue or intensify their training throughout childhood and adolescence [6,7]. 

Elite athletes’ health and performance are strongly affected by their dietary habits. Typically, they require above-average energy and macronutrient intakes to meet their daily energy expenditure and enable them to continue training, maintain their performance and recover from injury [8]. In particular, such athletes need adequate energy availability (EA) to sustain high performance levels and protect their long-term health; however, the efforts of some athletes, particularly female rhythmic gymnasts, to maintain a lean body increase the risk of them failing to meet nutritional requirements for health [9]. Insufficient caloric intake and/or excessive energy expenditure result in relative energy deficiency [10]. Low energy availability (LEA) refers to a state in which the total energy intake minus the energy expended in exercise is insufficient to cover energy needs for basic physiological processes [11]. The current LEA cut off for physically active females is <30 kcal/kg fat-free mass (FFM)/day [12]. For this athlete group, optimal EA is considered >45 kcal/kg FFM/day and sub-clinical EA is 30–45 kcal/kg FFM/day [13]. LEA may affect the often-immature musculoskeletal system of gymnasts, increasing the risk of injury to growth or articular cartilage and bones, and its impact on this and other systems gives rise to wider concerns about the risk of health problems including injury [14]. Further, excessive weight loss during growth has negative effects on body and bone composition, and LEA may affect the female reproductive system, delaying menarche and/or resulting in irregular menses [15,16].

Notably, several studies have indicated that many gymnasts aspire to attain a “perfect” body, characterized by specific body proportions with unrealistically low body weight and level of body fat [17,18,19]. As they age, it is not uncommon for gymnasts to repeatedly reduce their nutrient intake [20]. In this context, there is a clear need to monitor both energy and nutritional needs of this population of young athletes, to ensure proper growth and development [15]. Given all this, the aim of this study was to investigate body composition, dietary intake, and the risk of LEA in elite-level competitive rhythmic gymnasts. Our hypothesis was that teen gymnasts do not consume as much energy as they need each day.

## 2. Materials and Methods

The study population consisted of 30 elite gymnasts, whom we divided into two groups by age: pre-teen (9- to 12-year-olds: 10.51 ± 1.07 years) (*n* = 17) and teen (13- to 18-year-olds; 15.87 ± 1.43 years) (*n* = 13). The age cut-off corresponds to the age of transition from primary to secondary education in our setting when patterns of training tend to change. All gymnasts and their parents were given information about the objectives of the study, and before inclusion in the study, the gymnasts assented, and their parents gave written informed consent. The Ethics Committee of the University of Leon approved all procedures, and the study was conducted in compliance with the principles for clinical research of the World Medical Association Declaration of Helsinki. 

In general, gymnasts in the pre-teen group had five training sessions a week, with a total of 3 h/day, while the teens completed six training sessions a week, with a total of 4 h/day. All of the participants were in the national team of the Spanish Royal Gymnastics Federation and were considered elite gymnasts, having been selected to participate in national and European championships. Specifically, there are ten rhythmic gymnastics competitions a year in which they were eligible to compete.

The study was conducted during a training camp at Leon’s High Performance Centre (set up by the National Sports Council, part of the Spanish Ministry of Culture and Sports). During this event, the Royal Spanish Gymnastics Federation held technical tests, in front of an international panel of judges, as well as the technical director of the national team, for the gymnasts who were candidates to participate in upcoming international competitions. 

### 2.1. Body Composition

The anthropometry and body composition of elite gymnastics was measured before a routine training session in the middle of the competition season (January). Measurements were carried out after overnight fasting of at least 10 h and before the warm-up session. 

Body weight (BW) was measured to the nearest 0.01 kg using a digital scale (Seca 703, 02760 Azcapotzalco, Ciudad de México, México) with participants wearing T-shirts and gym shorts. Height was determined to the nearest 0.1 cm with a portable stadiometer (Detecto D52, United States). Body mass index (BMI) and BMI z-scores were calculated using WHO reference data for 5- to 19-year-olds [20]. Using a flexible tape, waist circumference (WC) was measured (in cm) at the end of a normal exhalation at the smallest circumference between the thorax, and the hip circumference (HC) was measured (in cm) at the largest circumference around the trochanters. From these values, the waist-to-hip ratio (WHR) was calculated. These anthropometric measurements were all taken by the same internationally certified anthropometrist (International Society for the Advancement of Kinanthropometry [ISAK] level 3, certificate number: #636739292503670742) following the ISAK protocol [21]. All measurements were taken twice to assess the reliability of the procedure. If the difference between the results exceeded 1% for an individual perimeter, a third measurement was taken. The mean of the measurements taken was recorded [21]. After anthropometry, body fat (BF), fat-free mass (FFM), bone mineral content, water intra and extracellular content, and muscle mass were assessed via bioimpedance analysis (Inbody 230, Microcaya, Bilbao, Bizkaia, Spain).

### 2.2. Nutrition Analysis

To assess participants’ dietary intake, they and/or their parents completed a 7-day food diary using forms provided for that purpose on the day the anthropometric measurements were taken. These forms asked for information about what participants consumed each day: amounts, proportions of ingredients, methods of preparation, and so forth [22]. Each of the forms had an initial instructions section to guide participants throughout the week-long process. Further, a well-trained interviewer, blinded to the anthropometric and laboratory results, interviewed the gymnasts and their parents the following day to confirm the information reported in the food diary. Subsequently, the same researcher conducted the nutritional analysis. 

The information gathered about intake of foods, including quantities/portion sizes, and cooking processes as well as any dietary supplements (for example, multivitamins) was processed using the Nutriber V.1.1.1.R5 software package (Funiber, Barcelona, Spain) to calculate total energy and fat intakes, and intakes of specific fatty acids, cholesterol, vitamins, and minerals. Food diaries combined with 24 h recall met the condition of being a simple, rapid, and inexpensive method to analyze dietary intake. 

The recommended daily intakes for macronutrients were 1.6 to 2 g/kg/day of proteins and 6 to 10 g/kg/day of carbohydrates, and further, 20 to 35% of total energy intake should be from fat [8,23]. Nutrient intake data were compared with the Spanish recommendations by gender and age [24,25] and recommendations for child and adolescent athletes [26,27]. 

### 2.3. Physical Activity-Related and Basal Energy Expenditure

Participants were instructed in how to secure the ActiGraph accelerometer (GT3x or GT3x+; ActiGraph LLC, Pensacola, FL, USA) under their clothing above their right hip, using an elastic waistband. They were asked to wear the accelerometer throughout their waking hours, except during water-based activities, and additionally to wear it during sleeping periods, unless they found that it was uncomfortable and interfered with their sleep. Further, participants were instructed to record any relevant incidents (e.g., problems with the accelerometer or disruption to their routine) on a sheet provided for that purpose. The accelerometer was used over 4 days (3 training days and 1 day off) [28]. The total amount of physical activity performed was expressed as the sum of recorded counts per day divided by the total wear time per day in minutes. The ActiGraph software computes physical activity-related energy expenditure using the equation of Ekelund et al. for children and adolescents [29]. On the other hand, basal metabolism was estimated according to the reference data of the World Health Organization (WHO) [20].

### 2.4. LEA Calculation

Energy availability (EA) refers to the total energy available for physiological processes after the energy expended in exercise has been subtracted from the dietary intake [11]. As described above, LEA occurs when there is a mismatch between energy intake and exercise, leading to insufficient energy to support normal physiological functions [11]. The current LEA cut off for physically active females is <30 kcal/kg fat-free mass (FFM)/day [20]. Moreover, for this athlete group optimal EA is consider >45 kcal/kg FFM/day, sub-clinical EA is 30–45 kcal/kg FFM/day [20], and extreme LEA when EA is <10 kcal/kg FFM [13]. Although these cut offs were estimated on female athletes over 18 age, there are some manuscripts that use them in under-18 athletes [30,31].

### 2.5. Statistical Analyses

IBM SPSS version 20 (Armonk, NY: IBM Corp) was used for statistical analysis of the data and GraphPad Prism 6 software (GraphPad Software, Inc., San Diego, CA, USA) for plotting the graphs. Descriptive statistics were calculated using the mean as a measure of central tendency and standard deviation as a measure of dispersion. Kolmogorov–Smirnov tests confirmed that the data were normally distributed in all groups. To assess whether differences between the two groups were significant, all the variables were analyzed using a univariate test using the category (pre-teen vs. teen) as a fixed factor. Finally, a chi-square test of prevalence was performed to examine the relation between LEA incidence and study group (pre-teen vs. teen). *p*-values < 0.05 were considered statistically significant.

## 3. Results

### 3.1. Body Composition

Table 1 presents the anthropometry and body composition data by group. As would be expected, the teens had significantly higher height, body weight, BMI, waist and hip circumference, and WHR (*p* < 0.05). Likewise, the teens showed significant more absolute muscle mass, fat mass, FFM, bone mineral content, and intracellular and extracellular water content (*p* < 0.005). On the other hand, there were no significant differences in the percentage of fat mass, FFM, or bone mineral content (*p* > 0.05).

The comparisons made between the mean weight and height of the gymnasts and the WHO reference data [20] indicate that the gymnasts in this study have lower than the average values for both parameters. 

### 3.2. Nutrition Survey

**Figure 1:**
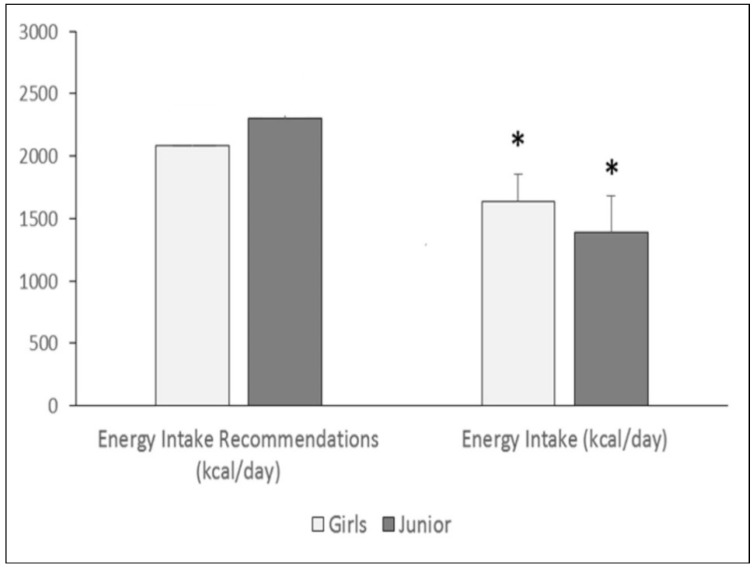
Recommended and measured average energy intake by age group (pre-teen vs. teen), compared to recommendations [25] (* *p* < 0.05 considered significant).

The results concerning participants’ nutrient intake indicate significant differences with respect to Spanish recommendations for pre-teens and teens. Specifically, the average energy intake was lower than the recommendations for their age in both groups (Figure 1), and notably, energy intake fell from 1640 kcal/day in 9- to 12-year-olds to 1388 kcal/day in 13- to 18-year-olds.

Table 2 shows energy, macronutrient intakes, and percentage of Recommended Dietary Allowance (RDA) by group (pre-teen and teen). When energy intake is calculated according to free-fat body mass, it is observed that the pre-teens’ (*n* = 17, 100%) intake was above the recommended value of 40 kcal/kg/day (60.04 ± 12.55 kcal/FFM/day), while that of the teens was markedly lower (29.70 ± 8.05 kcal/FFM/day) (Table 2). Among the older group, eleven gymnasts (75%) were identified as being at increased risk of LEA.

The distribution of the daily energy intake across the macronutrients indicates that both groups consume less than the recommended amount of carbohydrates (49.4 ± 7.20% in pre-teens, 48.9 ± 14.5% in teens vs. 50–55% recommended) and more than the recommended amount of fat (37.0 ± 6.6% in pre-teens, 32.8 ± 10.0% in teens vs. 30% recommended); no significant differences being observed between the two groups of gymnasts in this regard. On the other hand, the carbohydrate intake according to BW was 7.4 ± 1.5 g/kg BW/day in the pre-teens and 3.7 ± 1.3 g/kg BW/day in the teens (Table 3), this suggesting that the older group had an intake deficit considering the intensity of their workouts and the RDAs [25]. 

Given that the study participants are athletes who are still growing, their recommended protein intake is 2 g/kg BW/day. The pre-teen group consumed an average of 2.5 g/kg BW/day, whereas the teen group had an intake of 1.5 g/kg BW/day. Fiber intake was similar in the two groups (13.1 ± 4.1 g vs. 10.9 ± 1.7 g), but only covered the recommended minimum amount of 10 g/1000 kcal in the younger group.

The mineral and vitamin content of participants’ diets, shown in Table 4, reached values close to the recommendations. Athletes’ median intakes met or exceeded the RDA for all vitamins, except for folate, retinol, vitamin D, and E. Further, in both groups, intake of K, Ca, P, Fe, Zn, and I was lower than the dietary recommendations for girls 10–12 years (pre-teens) and 13–19 years (teens) [25]. 

**Figure 2:**
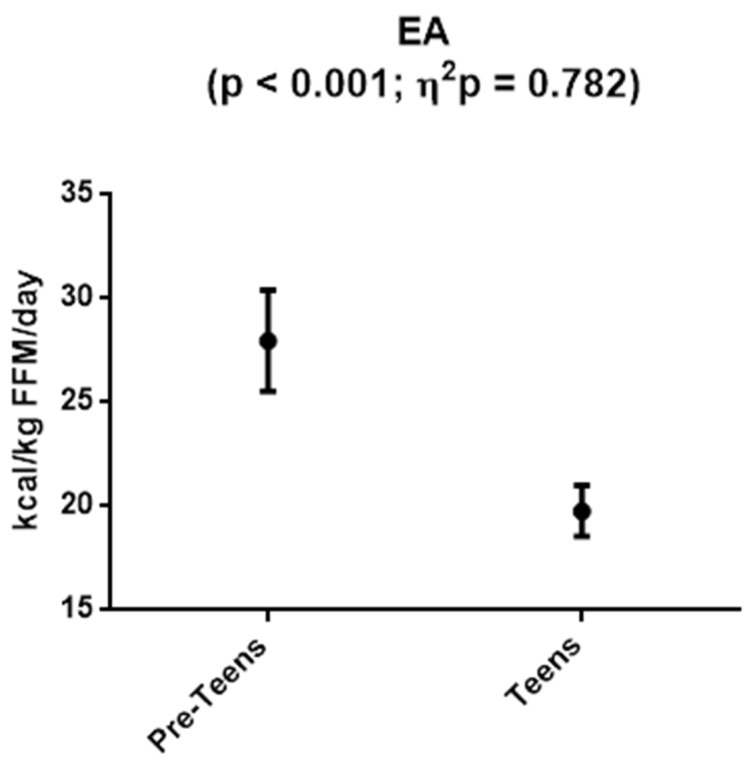
Energy availability values in pre-teen and teen gymnast groups. Data are expressed as mean ± standard deviation. *p*: *p*-value for differences between groups by univariate test (*p* < 0.05 being considered significant).

Figure 2 presents EA data for both groups. In this case, pre-teens had significantly higher EA values than teens (27.92 ± 2.43 vs. 19.73 ± 1.23 kcal/Kg FFM/day, respectively; *p* < 0.001).

Table 5 presents the prevalence of LEA in each study group. There were significant differences between groups in the distribution of cases (*p* = 0.005). While EA was sub-clinical in the pre-teens in 70.6% of cases, it was not adequate in any of the teens. Moreover, 100% of the teens group and 29.4% of the pre-teens were classified as having severe LEA (see Figure S1).

## 4. Discussion

The main finding of this study is that teenage gymnasts do not consume as much energy as they need each day, which explains their low body weight, as well as posing a risk for their performance and future health. In addition, the nutrient intake observed differs significantly from the Spanish recommendations for children and adolescents of their age. Notably, in both groups, the average energy intake was lower than recommended for their age.

This study is significant as it analyzes the body composition and dietary intake of a cohort of understudied athletes in Spain. It is very important to stress that this is a group of gymnasts who compete at a very high level; all are considered “elite”, and some are national and international champions. The athletes studied have a body composition typical of gymnasts, lower dietary intake than their peers and compared to recommendations for athletes [26,27], and a high prevalence of increased risk of LEA that might have a medium- long-term impact on their performance and health [32,33,34,35,36,37,38,39,40].

Relatively few studies have focused on anthropometric parameters and body composition in rhythmic gymnasts [4,17,41,42,43], but our study population presents characteristics typically associated with very intensive training [44]. Regarding body weight, the teens had significantly higher body weight and height than the pre-teens; nonetheless, in line with other research, the data indicate that they are smaller than their non-athlete peers [19]. The low body weight observed in our gymnasts is consistent with a short-term energy deficit. Moreover, their BMI values were close to the lower limit of the normal range established by the WHO (18.50 to 24.99 kg/m^2^—15th to 85th percentile) [45], indicating that underweight is a major concern in this cohort of athletes. In contrast, other studies with rhythmic gymnasts have reported higher values for BMI and percentage fat mass than those observed in both of our groups [4,31,46]. This may be because the gymnasts in our study were mostly younger and performed at a higher level than those in other studies.

Some authors have indicated that gymnasts have decreased adipose tissue and delayed skeletal maturation and pubertal development [16,47]. This is attributed to gymnasts generally being exposed to high levels of physical and psychological stress and subjected to many hours of training from preadolescence [42]. Moreover, athletes’ motivation to maintain a low weight to achieve particular results drives them to maintain a thin somatotype [41], which is evidently associated with a body aesthetic characterized by low fat mass percentages. Due to the major role of body shape and appearance in this type of sport, athletes often limit their energy intake and/or increase their energy expenditure through excessive exercise training to achieve body composition goals, meaning that they fail to satisfy their energy requirements [16], and this may elevate the risk of LEA [8].

The energy intakes of the gymnasts in our study were lower than the Spanish energy intake recommendations. An energy deficit is commonly reported in athletes [48,49], but it is extremely difficult to accurately assess energy needs in young athletes [49,50], and self-reported dietary records are limited. It appears that LEA in adolescent athletes undertaking heavy training is common [16,51]. 

Inadequate energy consumption represents a powerful stimulus to the endocrine system, leading to detrimental effects that predominantly involve the reproductive system (primary or secondary amenorrhea, chronic anovulation) and, subsequently, bone metabolism and bone mass (osteopenia and osteoporosis) [16,51,52]. In particular, various studies have reported that athletes who have engaged in intensive training and participated in competitive sports during childhood may experience a delay in menarche, especially sports that emphasize a lean body physique [53]. Our rhythmic gymnasts demonstrate lower body weight and fat than their population average [18], and 92% of the group 13- to 18-year-olds had not their first menstruation at the time of the study. These results are consistent with those of another study that found that young rhythmic gymnasts who trained intensively had menarche delayed by at least 2 years compared to other athletes and their non-athlete peers, increasing the likelihood of secondary amenorrhea [54]. In that study, almost all the athletes eventually, though in some cases with a marked delay, achieved regular cycles, and the authors suggested that this implied a minimal impact on fertility and bone density. Nonetheless, a disruption of menstruation might increase the risk of developing a range of health problems. 

Further, female athletes with insufficient energy intake to balance the energy that they expend during exercise are at risk of LEA, which is the cornerstone of a condition called the relative energy deficiency in sport (RED-S) [10,33,55,56,57]. This RED-S is the result of LEA (with or without an eating disorder) whose consequences can alter many physiological systems, including menstrual function, bone health, immunity, protein synthesis, and psychological [58]. Management strategies should address the complex conditions underlying functional hypothalamic hypogonadism [16,53], though this concern is more applicable to older athletes than it is to the majority of our sample [59]. Notably, the decline in diet quantity observed in the teens compared to that of the pre-teens in this cohort is in line with teenage eating habits in general and with higher rates of disordered eating observed among teenagers [60], suggesting that coaches and parents should monitor this group particularly carefully. Other authors have also commented on the need to improve the diet of female athletes aged 13 to 18 [16,49]. In relation to this, low energy intake was observed in young female artistic gymnasts in Palma de Mallorca (Spain) [61]. Notably, despite the low energy intakes most blood parameters and micronutrient intakes remained within ranges considered normal. Unfortunately, no significant changes in the main characteristics of the gymnasts’ diet were observed after a nutritional intervention [61]. It is reasonable to suggest that many adolescent athletes require additional knowledge, skills, and support to develop a healthy lifelong relationship with food.

Concerning specific dietary components, numerous researchers and scientific societies [8,23] have indicated their positions on the amount of carbohydrates that athletes should consume depending on their sports specialties. There is little evidence to suggest that the utilization of carbohydrate in adolescents differs substantially from that of adults [50]. Carbohydrate recommendations based on body weight rather than percent of total calories are thought to best reflect athletes’ needs and range from 3 to 12 g/kg BW, depending on exercise load [49,62]. The gymnasts in our study exhibit different characteristics in that the younger group consume an appropriate amount of carbohydrates (pre-teens: 7.4 g/kg BW/day), while the older group eat half the recommended amount (teens: 3.7 g/kg BW/day). Additionally, both groups eat less fiber than recommended; suggesting that information should be provided to gymnasts on the quantity and quality of carbohydrates to help them to eat an adequate diet.

In assessing protein requirements, total energy intake is also an important consideration, since an inadequate energy intake will cause proteins to be used as a substrate for energy, potentially reducing its availability for its primary functions [63]. Protein recommendations have increased and range from 1.2 to 2.0 g/kg BW/day depending on the type and amount of physical activity [49]. Furthermore, it has been estimated that protein intakes of 1.5 g/kg BW/day are required for nitrogen balance in young sprinters [64] and sufficient for most young athletes (for performance and growth). A protein intake of ~1.5 g/kg BW/day should be sufficient to replace any exercise-induced amino acid oxidative losses, enhance whole-body net protein balance, and support the normal growth and development of adolescent athletes [64]. Based on this criterion, gymnasts in both groups in our study consumed enough protein, but there were significant differences, the pre-teens consuming more protein (2.6 g/kg vs. 1.6 g/kg in teens). Indeed, average protein intakes exceeded the upper recommendation for performance in the younger group but not in the 13- to 18-year-olds, a situation similar to that described by other authors [49,65].

Regarding minerals and vitamins, our gymnasts did not meet the micronutrient recommendations in most cases respect to RDA for the general population in Spain for girls 10–12 years (pre-teens) and 13–19 years (teens) [25]. Notably, however, teens tended to have lower intakes than the pre-teens, indicating that the older group is at a higher risk of not ingesting enough of some micronutrients.

Specifically, iron and folate were identified among nutrients whose intakes often did not meet the RDA in the teen group. Deficiencies in either of these nutrients can promote anemia, which can cause fatigue and suboptimal athletic performance [66]. In general, young female athletes are at a higher risk of iron deficiencies than their sedentary peers, because of their greater requirements for intense training, and their male counterparts, because of lower energy intakes and losses from menses (in such cases). Diet assessments in other athletic populations have also found inadequate intakes for iron and/or folate in young female athletes [67].

In addition, very low intakes of calcium and vitamin D were observed. Gymnasts in both groups failed to meet RDAs for both of these micronutrients, which is in agreement with previous studies conducted on elite gymnasts [55,61,68]. Vitamin D deficiency and insufficiency is common in children worldwide. A high prevalence of vitamin D deficiency and insufficiency has been documented in Europe, China, India, the Middle East, and South America, where foods are not fortified with vitamin D [69]. Though also an issue described in the Spanish population in general, this is particularly important in children and adolescents who are in the growth phase and, particularly, in those who undertake intense training. Low intakes of vitamin D and calcium could be related. Specifically, they are both consistent with a low intake of dairy products. In our study, we did not measure serum levels of vitamin D, and hence, we do not know whether levels were deficient. Notably, however, Lovell et al. (2008) [69] evaluated the vitamin D status of professional female gymnasts, and they suggested that the vitamin D and calcium status should be monitored in athletes in gymnastics and other sports conducted indoors such as volleyball and basketball. It seems clear that more studies are needed to more accurately monitor vitamin D status in adolescent athletes. 

To achieve optimal nutritional support for physiological function and performance, it is important to consider the macronutrient content of the diet. Indeed, low carbohydrate availability has been linked to poorer performance [70]. LEA increases the risk of injury and illness and hence may have an impact on performance due to lost training time [71]. Further, it has been suggested that long-term LEA may negatively affect sports performance through indirect mechanisms, such as impairment of recovery and losses of muscle mass and function [72,73]. In line with this, Tornberg et al. [74] suggest that achieving or maintaining a lower body weight through long-term LEA is likely to negatively affect health and performance. Nonetheless, relatively few studies have investigated the impact of LEA on performance [74] and, to our knowledge, none have explored this question in gymnasts. 

In this context, it has been suggested that, in line with annual training programs for athletes, their dietary intake should be varied over time, a strategy called “nutrition periodization”, to optimize their condition and performance. That is, even though optimal EA is crucial for good health and optimal performance, periods of subclinical LEA may be required to reach the physique desired [74].

The potential negative effects of LEA and associated conditions on sporting performance [12,75] and the limited evidence regarding EA in gymnastic disciplines indicate the need for more research in this area. Nonetheless, it is already evident, as Logue et al. noted in a recent review [76], screening and interventions for LEA risk are needed to protect athletes’ health and performance [75]. Coaches can play a crucial role in the early identification of athletes at risk of LEA. Thus, it is necessary to establish educational initiatives to educate coaches and athletes about the importance of developing adequate dietary strategies that ensure the consumption of sufficient calories to support training and improve performance [77].

The first study to provide career-long data on the periodization of body composition and nutrition of a female Olympic-level athlete, a middle-distance runner, was reported recently [78]. In the general preparation phase, optimal AE was prioritized, and the athlete’s body weight was slightly higher (∼2.1%) as was her body fat percentage (∼17.8% as estimated using the Yuhasz method) than in the competition phase. In contrast, in the competitive preparation phase, the target was to achieve optimal body composition and race weight for the competitive season (May to August) over 6 to 8 weeks through moderate caloric restriction (−300 kcal/day), with a lower intake of snacks, energy-dense foods (sweetened or fatty), and carbohydrate-rich foods on days of less intense training, while maintaining adequate protein intake (2.0–2.5 g/kg/day). Notably, this case study illustrates how optimal physique could be achieved at key moments in an athlete’s calendar while protecting a female athlete’s long-term hormonal and bone health. 

The main limitation of the study is the relatively small size of the sample. Nonetheless, considering the high level of performance of the athletes, it is not easy to find large samples to carry out this kind of study. The use of self-report questionnaires is another limitation, as they could limit the accuracy and validity of the dietary assessment method, in particular, for measuring micronutrient intake. Likewise, no parameter was included to determine the degree of development of the gymnasts, including the control of the menstrual cycle in teenage gymnasts. The main strength of this study is the inclusion of only elite gymnasts, all of whom are members of a team that competes in national and international tournaments. 

## 5. Conclusions

Our research is novel in that, to our knowledge, it provides the first assessment of body composition and dietary intakes and the risk of LEA in elite-level competitive rhythmic gymnasts in Spanish preadolescent and adolescent elite rhythmic gymnasts. 

The main conclusion of our work is that teenage gymnasts do not consume as much energy as they need each day, which explains their body weight, as well as putting them at a higher risk of developing LEA that may negatively impact their future health and performance. In particular, athlete support staff should underline the need for an appropriate carbohydrate intake to balance exhaustion against excessive consumption, since low carbohydrate consumption can compromise performance both in training and in competition. They should also encourage the intake of dietary sources of vitamin D and calcium. 

## Figures and Tables

**Table 1:** General characteristics and body composition by bioimpedance of the participants.

Variables	Pre-Teens (9–12 Years), *n* = 17	Teens (13–18 Years), *n* = 13	*p*	η2p
Height (cm)	136.2 ± 11.1	159.8 ± 4.6 *	<0.001	0.597
Body weight (kg)	29.5 ± 6.4	46.8 ± 5.7	<0.001	0.652
BMI	15.8 ± 1.6	18.0 ± 1.8	0.003	0.300
BMI z-score	−0.80 ± 0.74	−1.15 ± 1.18	0.348	0.035
Waist circumference (cm)	54.9 ± 3.7	66.0 ± 4.6	<0.001	0.592
Hip circumference (cm)	67.9 ± 5.4	82.8 ± 5.6 *	<0.001	0.637
WHR	0.81 ± 0.03	0.82 ± 0.02	0.018	0.205
Muscle mass (kg)	12.5 ± 2.4	22.5 ± 2.3 *	<0.001	0.697
Muscle mass (%)	44.1 ± 2.4	46.8 ± 3.0	0.017	0.207
Fat mass (kg)	4.1 ± 1.6	7.1 ± 3.40 *	0.003	0.294
Fat mass (%)	14.0 ± 3.8	14.3 ± 5.6	0.763	0.004
Fat-free mass (kg)	25.4 ± 5.6	41.2 ± 3.9 *	<0.001	0.698
Fat-free mass (%)	86.2 ± 3.8	85.7 ± 5.6	0.763	0.004
Bone mineral content (kg)	1.4 ± 0.2	2.4 ± 0.3 *	<0.001	0.719
Bone mineral content (%)	4.9 ± 0.3	5.1 ± 0.8	0.625	0.010
Extracellular water content (kg)	7.1 ± 1.5	11.4 ± 1.8	<0.001	0.686
Intracellular water content (kg)	11.6 ± 2.5	18.8 ± 1.1	<0.001	0.699

Data are reported as mean ± standard deviation. BMI: body mass index; WHR: waist-to-hip ratio. *: *p*-value for differences between groups by one-way analysis of variance (*p* < 0.05 being considered significant).

**Table 2:** Energy, macronutrient intake and percentage of Recommended Dietary Allowance (RDA) in pre-teen (9- to 12-year-old) and teen (13- to 18-year-old) gymnast groups (*n* = 17 and *n* = 13, respectively).

	Total Intake	% RDA ^†^	% Below RDA	% Met RDA	% Exceeded RDA
Energy (kcal)					
Pre-teen	1640 ± 212	78.7	38.1	62.0	0.0
Teen	1388 ± 295	60.36	85.71	14.29	0.00
Water (mL)					
Pre-teen	1299 ± 244				
Teen	1303 ± 307				
Protein (g)					
Pre-teen	70.7 ± 13.9	208.1	0.00	0.00	100.0
Teen	73.6 ± 8.3	160.1	0.00	0.00	100.0
Protein% kcal (10–15%)					
Pre-teen	17.25 ± 3.38		0.0	20	80.0
Teen	21.2 ± 2.4 *		0.0	0.0	100.0
Fat (g)					
Pre-teen	67.4 ± 10.2	88.2	20.0	80.0	0.0
Teen	50.6 ± 15.4 *	60.04	85.71	14.29	0.00
Fat% kcal (20–35%)					
Pre-teen	37.0 ± 6.6		0.0	40.0	60.0
Teen	32.8 ± 10.0		0.0	57.1	42.9
SFAs (g)					
Pre-teen	19.8 ± 5.2	107.2	13.33	73.33	13.33
Teen	16.5 ± 8.2 *	80.85	57.14	28.57	14.29
MFAs (g)					
Pre-teen	24.8 ± 5.7	53.6	86.67	13.33	0.00
Teen	20.1 ± 5.9 *	39.26	100.0	0.00	0.00
PFAs (g)					
Pre-teen	6.6 ± 1.4	57.2	93.33	6.67	0.00
Teen	5.3 ± 1.1	41.10	100.0	0.00	0.00
Cholesterol (mg) < 300 g					
Pre-teen	235.0 ± 93.6		-	76.60	19.10
Teen	249.4 ± 56.1		-	83.33	16.7
Carbohydrate (g)					
Pre-teen	202.6 ± 29.9	70.1	73.33	26.67	0.00
Teen	169.6 ± 50.4	53.19	85.71	14.29	0.00
Carbohydrate% kcal (>50%)					
Pre-teen	49.4 ± 7.20		60.0	40.0	0.00
Teen	48.9 ± 14.5		57.1	42.9	0.00
Fiber (g)					
Pre-teen	13.8 ± 4.1	55.4	100.0	0.00	0.00
Teen	10.9 ± 1.7	43.43	100.0	0.00	0.00

Intakes are presented as mean ± SD. BW, body weight; kcal, kilocalories; SFAs, saturated fatty acids, MUFAs, monounsaturated fatty acids; and PUFAs, polyunsaturated fatty acids. * Differences by independent *t*-test significant at *p* < 0.05 taking the pre-teens as the reference group. Percentage of sample by whether gymnasts met the RDA (intakes of ≤75%, between 76–125%, and ≥126% of the RDA being classified as “below”, “met” and “exceeded” the recommendations respectively). ^†^ RDA for the general population in Spain [25].

**Table 3:** Energy, macronutrient intakes per body weight in pre-teen and teen gymnast groups.

	Pre-Teen (9–12 Years), *n* = 17	Teen (13–18 Years), *n* = 13
Energy (kcal/kg FFM)	66.9 ± 9.5	33.6 ± 8.0 *
Protein (g/kg BM)	2.6 ± 0.6	1.6 ± 0.3 *
Carbohydrate (g/kg BM)	7.4 ± 1.5	3.7 ± 1.3 *
Fat (g/kg BM)	2.5 ± 0.6	1.2 ± 0.4 *

Intakes are presented as mean ± SD. * Differences by univariate *t*-test significant at *p* < 0.05 taking the pre-teens as the reference group.

**Table 4:** Micronutrient intakes and percentage of RDA values in pre-teen and teen gymnast groups.

		Pre-Teen (9–12 Years) (*n* = 17)	% RDA ^†^	Teen (13–18 Years) (*n* = 13)	% RDA
Minerals	Na (mg)	1617 ± 488	-	1612 ± 390	-
	K (mg)	1999 ± 355	64.5	2184 ± 614	62.4
	Ca (mg)	831.2 ± 189.8	63.9	840.9 ± 267.5	64.6
	Mg (mg)	231.1 ± 29.6	77.0	252.8 ± 70.4	76.6
	P (mg)	1134 ± 188	94.5	1088 ± 213	90.6
	Fe (mg)	13.5 ± 4.6	75.0	11.1 ± 2.6 *	61.7
	Cu (mg)	0.7 ± 0.1	-	0.6 ± 0.1	-
	Zn (mg)	7.9 ± 4.1	52.7	10.5 ± 5.0	70.0
	Cl (mg)	1132 ± 540	-	1262 ± 336	-
	Mn (mg)	2.4 ± 1.7	-	4.9 ± 4.8	-
	Se (µg)	52.0 ± 15.8	115.6	60.0 ± 14.8	120.0
	I (µg)	43.4 ± 17.0	37.7	80.1 ± 30.2	69.7
Vitamins	B1 (mg)thiamine	1.3 ± 0.5	144.4	1.2 ± 0.2	120.0
	B2 (mg)riboflavin	1.7 ± 0.4	121.4	1.6 ± 0.5	106.7
	B6 (mg)	4.1 ± 1.4	256.3	1.7 ± 0.3 *	100.0
	B12 (µg)	7.3 ± 3.8	365.0	4.7 ± 0.9 *	235.0
	Folate (µg)	222.4 ± 72.4	74.1	190.8 ± 49.3	47.5
	Niacin (mg)	19.4 ± 5.5	129.3	20.7 ± 2.6	121.8
	C (mg)	83.8 ± 41.4	139.7	68.9 ± 44.9	114.8
	Pantothenic (mg)	4.0 ± 1.8	-	4.1 ± 1.1	-
	Biotin (mg)	7.8 ± 3.2	-	4.0 ± 1.6 *	-
	Retinol (µg)	670.6 ± 222.8	83.8	619.2 ± 364.7	77.4
	D (µg)	3.0 ± 2.9	20.0	3.7 ± 3.9	24.7
	E (mg)	7.8 ± 2.8	78.0	6.0 ± 1.9	52.2

Intakes are mean ± SD. Percent RDA for each athlete was calculated by dividing their intake by the established RDA for that age and gender and multiplying by 100. * Differences by univariant *t*-test significant at *p* < 0.05 taking the pre-teens as the reference group (*) ^†^ RDA for the general population in Spain [25].

**Table 5:** Low energy availability (LEA) prevalence of in each study group.

	Pre-Teen (9–12 Years) (*n* = 17)	Teen (13–18 Years) (*n* = 13)	*p*
Adequate EA	0 (0%)	0 (0%)	0.005
Subclinical EA	12 (70.6%)	0 (0%)	
LEA	2 (29.4%)	13 (100%)	
Extreme LEA	0 (0%)	0 (0%)	

Data are expressed as a per-centage of cases. Adequate EA: >45 kcal/kg FFM/day; Sub-clinical EA: 30–45 kcal/kg FFM/day; LEA: <30 kcal/kg FFM and extreme LEA: <10 kcal/kg FFM [13,20]. *p*: *p*-value for differences between groups by chi-square test (*p* < 0.05 being considered significant).

## Data Availability

The datasets used and/or analyzed during the current study are available from the corresponding author on reasonable request.

## Supplementary Materials

The following are available online at https://www.mdpi.com/article/10.3390/nu13062083/s1, Figure S1: Low energy availability (LEA) prevalence of in each study group.
