# Supplementary material for: Body Composition, Dietary Intake and the Risk of Low Energy Availability in Elite-Level Competitive Rhythmic Gymnasts
Source: Nutrients. 2021 Jun 18;13(6):2083. doi: 10.3390/nu13062083 (PMC8233987; doi:10.3390/nu13062083)
Supplement: Supplementary file 1 [file nutrients-13-02083-s001.zip › nutrients-1260353-supplementary.pdf]

nutrients

Supplementary

Body Composition, Dietary Intake and the Risk of Low Energy Availability in Elite-Level Competitive Rhythmic Gymnasts

María Villa [1], José Gerardo Villa [2], Jesus Seco-Calvo [2,3,*], Juan Mielgo-Ayuso [4] and Pilar S. Collado [2]

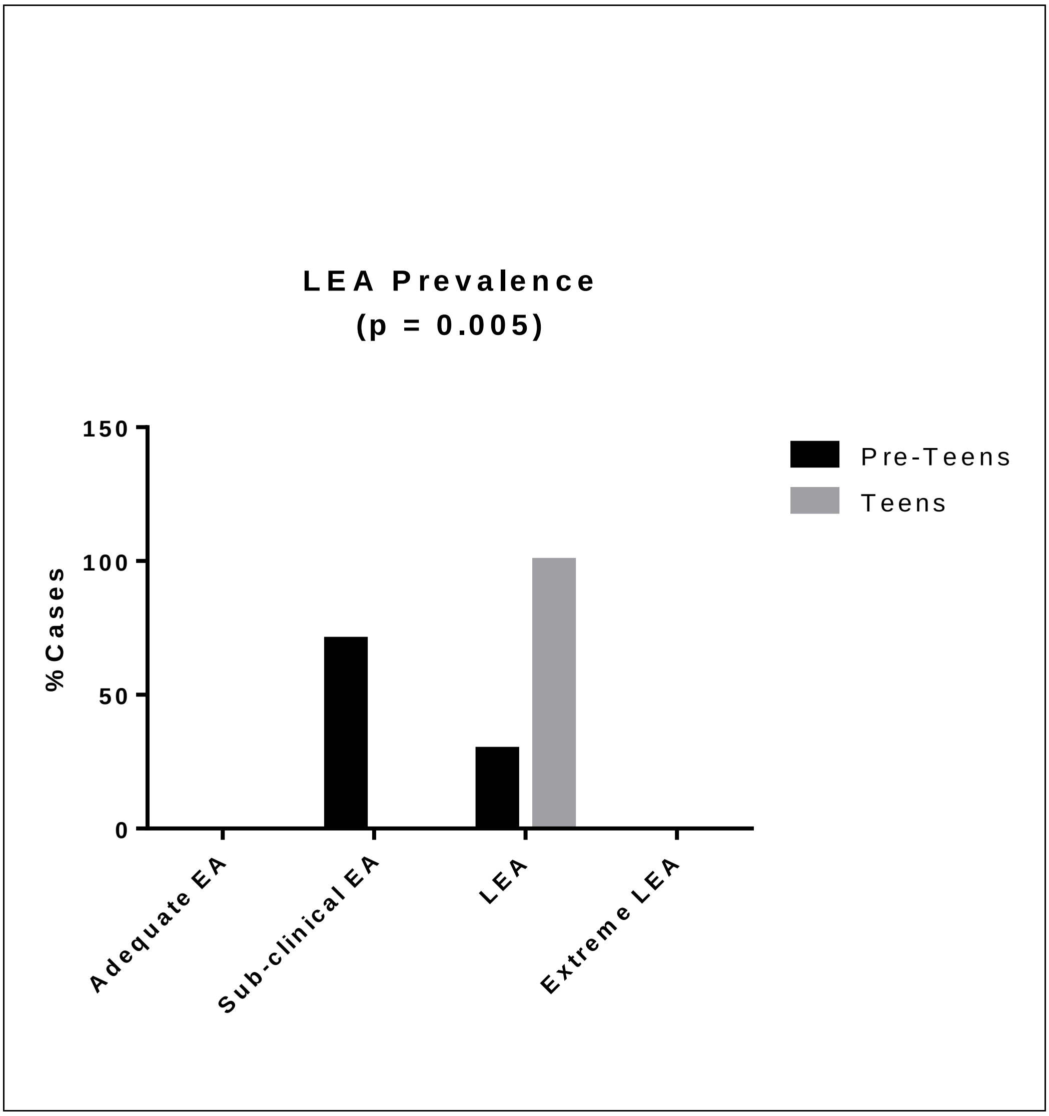

Figure S1. Low energy availability (LEA) prevalence of in each study group. Data are expressed as a percentage of cases. Adequate EA: > 45 kcal/kg FFM/day; Sub-clinical EA: 30–45 kcal/kg FFM/day; LEA: < 30 kcal/kg FFM and extreme LEA: < 10 kcal/kg FFM [13,20]. p: *p*-value for differences between groups by chi-square test ($p < 0.05$ being considered significant).
